# Urinary eicosanoid levels in early life and risk of atopic disease in childhood

**DOI:** 10.1016/j.jaci.2024.05.022

**Published:** 2024-06-01

**Authors:** Liang Chen, Nicklas Brustad, Min Kim, Yang Luo, Tingting Wang, Mina Ali, Nicole Prince, Yulu Chen, Su Chu, Sofina Begum, Kevin Mendez, Rachel S. Kelly, Ann-Marie Schoos, Morten A. Rasmussen, Javier Zurita, Johan Kolmert, Jakob Stokholm, Augusto Litonjua, Scott T. Weiss, Klaus Bønnelykke, Craig E. Wheelock, Jessica Lasky-Su, Bo Chawes

**Affiliations:** a Copenhagen Prospective Studies on Asthma in Childhood, Herlev and Gentofte Hospital, University of Copenhagen; b Faculty of Health and Medical Sciences, University of Surrey, Guildford; c Channing Division of Network Medicine, Brigham and Women’s Hospital and Harvard Medical School, Boston; d Department of Pediatrics, Slagelse Hospital; e Section of Microbiology and Fermentation, Department of Food Science, University of Copenhagen; f Unit of Integrative Metabolomics, Institute of Environmental Medicine, Karolinska Institutet, Karolinska University Hospital, Stockholm; g Department of Respiratory Medicine and Allergy, Karolinska University Hospital, Stockholm; h Division of Pediatric Pulmonary Medicine, Golisano Children’s Hospital, University of Rochester Medical Center.

**Keywords:** Atopic dermatitis, eicosanoids, COPSAC, VDAART, childhood asthma, type-2 inflammation

## Abstract

**Background:**

Eicosanoids are lipid mediators including thromboxanes (TXs), prostaglandins (PGs), and leukotrienes with a pathophysiological role in established atopic disease. However, their role in the inception of disease is unclear. This study aimed to investigate the association between urinary eicosanoids in early life and development of atopic disease.

**Methods:**

This study quantified the levels of 21 eicosanoids in urine from children from the COPSAC_2010_ (Copenhagen Prospective Studies on Asthma in Childhood 2010) (age 1 year, n = 450) and VDAART (Vitamin D Antenatal Asthma Reduction Trial) (age 3 years, n = 575) mother-child cohorts and analyzed the associations with development of wheeze/asthma, atopic dermatitis, and biomarkers of type-2 inflammation, applying false discovery rate of 5% (FDR5%) multiple testing correction.

**Results:**

In both cohorts, analyses adjusted for environmental determinants showed that higher TXA_2_ eicosanoids in early life were associated with increased risk of developing atopic dermatitis (*P* < FDR5%) and type-2 inflammation (*P* < .05). In VDAART, lower PGE_2_ and PGI_2_ eicosanoids and higher isoprostanes were also associated with increased risk of atopic dermatitis (*P* < FDR5%). For wheeze/asthma, analyses in COPSAC_2010_ showed that lower isoprostanes and PGF_2_ eicosanoids and higher PGD_2_ eicosanoids at age 1 year associated with an increased risk at age 1-10 years (*P* < .05), whereas analyses in VDAART showed that lower PGE_2_ and higher TXA_2_ eicosanoids at age 3 years associated with an increased risk at 6 years (*P* < FDR5%).

**Conclusions:**

This study suggests that early life perturbations in the eicosanoid metabolism are present before the onset of atopic disease in childhood, which provides pathophysiological insight in the inception of atopic diseases.

Eicosanoids are bioactive signaling lipids, including prostaglandins (PGs), thromboxanes (TXs), and leukotrienes (LTs), that possess pathophysiological roles in established atopic diseases.^1-3^ Prostaglandin D_2_ (PGD_2_) is released by mast cells and causes bronchoconstriction and inflammation in allergic asthma,^4,5^ whereas PGE_2_ is produced by airway epithelium and has broncho-protective and anti-inflammatory activities.^6^ Thromboxane A_2_ (TXA_2_) is released by platelets^7^ causing platelet aggregation^8^ and airway hyperresponsiveness.^7,9^ LTs are generated via 5-lipoxygenase^10^ and are potent bronchoconstrictive and proinflammatory mediators.^11,12^ Lastly, isoprostanes (IsoPs) are PG-like compounds formed via nonenzymatic free radical-initiated peroxidation of arachidonic acid^13^ and are elevated in airway inflammation and asthma.^14,15^

Eicosanoids can increase up to 100-fold in local concentration but are rapidly metabolized and excreted via the urine.^3,16,17^ Therefore, measurements of eicosanoids in blood are difficult, and the quantification of eicosanoids in urine is a more accurate assessment of whole body formation over a period of time. Thus, a previous longitudinal study showed that monitoring urinary eicosanoids could identify asthma with type-2 inflammation in adults and adolescents,^3^ which holds promise for a noninvasive approach for molecular phenotyping of asthma. However, there is a lack of studies investigating the role of eicosanoids in early life prior to onset of atopic disease, which could provide important pathophysiological insight and potentially novel targets for prevention and treatment.

In this study, we conducted the largest evaluation to date of urinary eicosanoid levels measured in early life in relation to the development of atopic diseases in children from the COPSAC_2010_ (Copenhagen Prospective Studies on Asthma in Childhood2010) and the VDAART (Vitamin D Antenatal Asthma Reduction Trial) birth cohorts.

## METHODS

### Study populations

The COPSAC_2010_ population-based, mother–child cohort of 738 pregnant women and their 700 children has previously been described in detail.^18-21^ The children underwent scheduled and acute care visits until age 10 years for close clinical phenotyping done exclusively by the COPSAC pediatricians.^18,19,22^

The VDAART mother–child cohort consists of 881 women with a family history of asthma, eczema, or allergic rhinitis and their 810 children. The children were monitored by telephone every 3 months and in-person annually for 6 years, during which offspring health, respiratory symptoms, and medications were assessed.^23,24^

**FIG 1.**
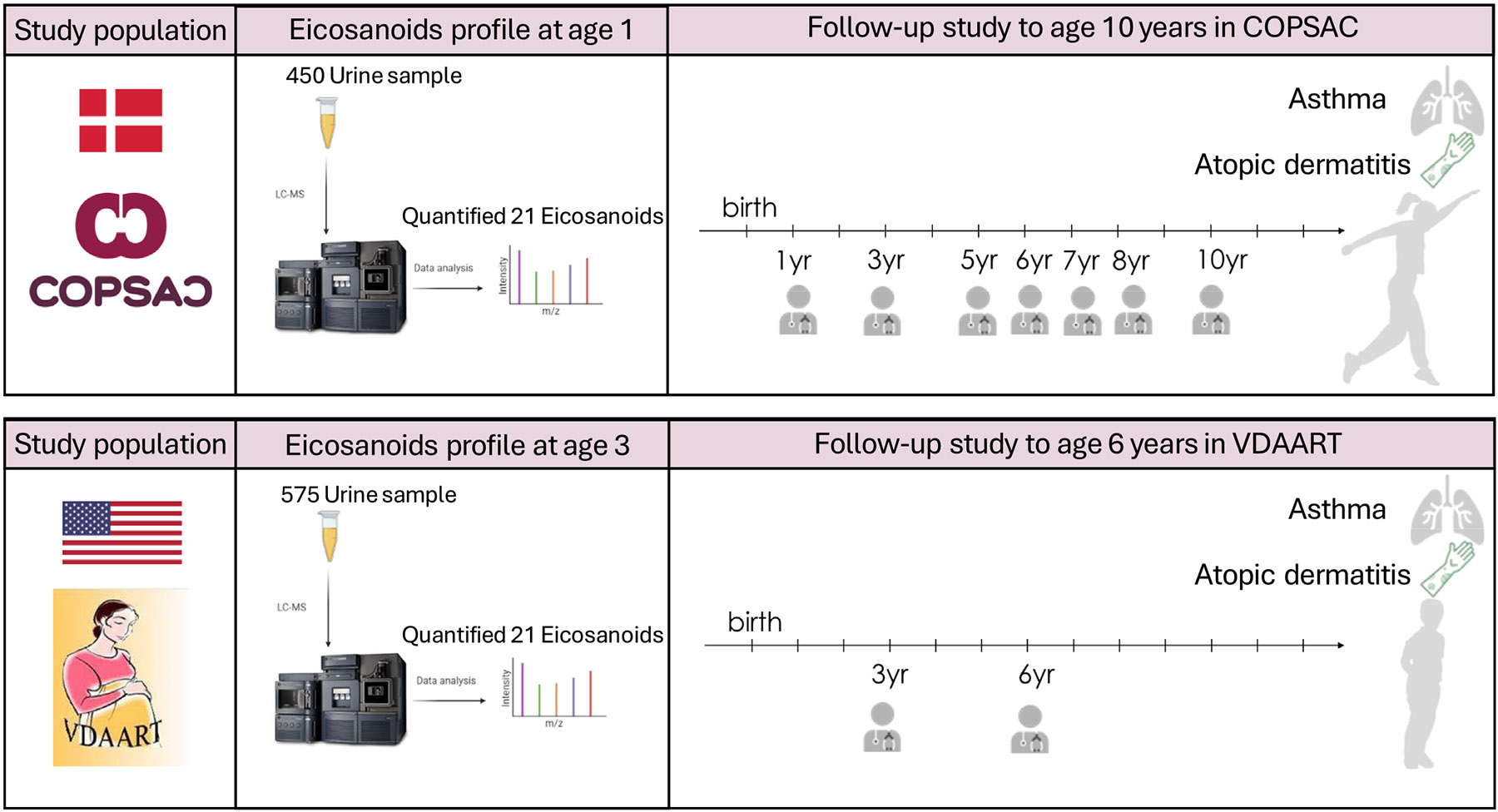
Overall study design. The study incorporates 2 independent cohorts: COPSAC_2010_ in Denmark, VDAART in USA. We quantified levels of 21 eicosanoids in urine from children participating in the COPSAC_2010_ (age 1 year, n = 450) and VDAART (age 3 years, n = 575) mother–child cohorts and analyzed associations with development of wheeze, asthma, AD, and biomarkers of type-2 inflammation.

An overview of the study is outlined in Fig 1.

### Data collection and clinical endpoints of COPSAC_2010_

#### Wheeze/asthma from 1 to 10 years.

##### Recurrent wheeze.

The diagnosis required a minimum of 5 episodes of diary-recorded and physician-verified troublesome lung symptoms lasting ≥3 consecutive days within the preceding 6 months or 4 weeks of consecutive symptoms.^18,19^

##### Persistent wheeze/asthma.

The diagnosis required (1) recurrent wheeze, (2) typical asthma symptoms, (3) need of shortacting inhaled beta-2-agonist, and (4) response to a 3-month trial of inhaled corticosteroids with relapse on cessation. The diagnosis “persistent wheeze” was used until age 3 years and “asthma” thereafter.^18,19^

#### Atopic dermatitis from 1 to 10 years.

The children were diagnosed according to Hanifin and Rajka’s criteria requiring presence of 3 of 4 major criteria and at least 3 of 23 minor criteria.^25^

### Data collection and clinical endpoints of VDAART

#### Wheeze/asthma.

Asthma was defined according to the composite of (1) asthma (parental report of physician-diagnosed asthma) and (2) recurrent wheeze (a parental report of recurrent wheeze in the child’s first 3 years of life) as detailed previously.^23,24,26^

#### Atopic dermatitis.

Parental report of physician’s diagnosis of eczema with rash in typical distribution as detailed previously.^23,24,26^

### Quantification of urinary eicosanoids

The COPSAC_2010_ urine samples were collected at age 1 year, whereas VDAART urine samples were collected at age 3 years. Urine samples from COPSAC_2010_ and VDAART were stored in −80°C prior to analysis. Urinary eicosanoids were quantified in 300 μL of urine following a single solid-phase extraction in 96-well format, adapted from the method by Gómez et al.^17^

### Data analysis

**FIG 2.**
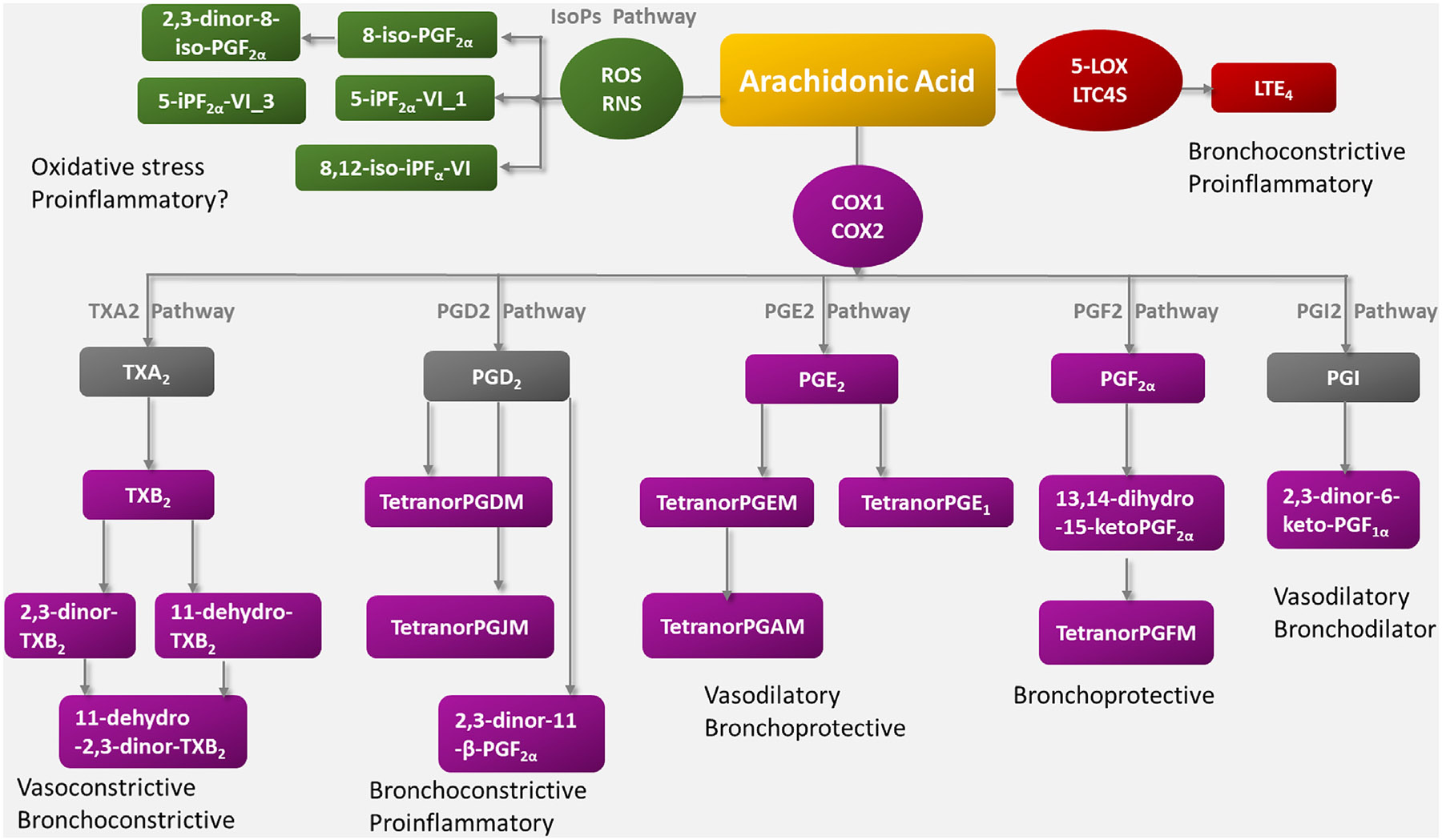
Schematic overview of arachidonic acid–derived eicosanoids following both enzymatic and nonenzymatic metabolism (*gray boxes*: these metabolites were not measured in this study). *RNS*, Reactive nitrogen species; *ROS*, reactive oxygen species.

The COPSAC_2010_ urinary eicosanoids data set included 450 children, whereas the VDAART data set included 575 children (Fig 2). Univariate linear and logistic regression analyses were employed to study the association between quantitative urinary levels of eicosanoids and the clinical outcomes, including wheeze/asthma, atopic dermatitis (AD), and type-2 inflammation markers (allergic sensitization [yes/no], and continuous assessments of total-IgE, fractional exhaled nitric oxide [Feno], and peripheral blood eosinophil count) as secondary clinical endpoints (for details, see the Methods section in this article’s Online Repository at www.jacionline.org). For repeated measurement outcomes such as yearly prevalence of persistent wheeze/asthma and AD, we used a general estimation equation model to compute the overall odds ratios with 95% CI. All regression models were adjusted for significant environmental determinants of eicosanoid levels. We applied a false discovery rate of 5% (FDR5%) to account for multiple testing.

First, we analyzed metabolites belonging to the same eicosanoid subpathways combined as composite variables by adding individual metabolites level, for example, combined TXA_2_s (c-TXA2s) = TXB_2_ + 11-dehydro-TXB_2_ + 2,3-dinor-TXB_2_ + 11-dehydro-2,3-dinor-TXB_2_. Second, we analyzed individual metabolites belonging to each of the 6 pathways: TXA_2_, PGD_2_, PGE_2_, PGF_2_, PGI_2_, and IsoPs in relation to the clinical endpoints. The subpathway classification and abbreviation of eicosanoid metabolites is outlined in Table E1 in this article’s Online Repository (available at www.jacionline.org). Third, we performed a principal component analysis (PCA) in COPSAC_2010_ and used PC1 and PC2 to explore the association between the profile of eicosanoids and clinic endpoints in the 2 cohorts separately.

All statistical analyses were performed using R (version 4.2.2; R Foundation, Vienna, Austria).

## RESULTS

### Baseline characteristics

A comparison of baseline characteristics among children with and without eicosanoid data showed significant differences in COPSAC_2010_ for sex (males: 59% vs 37%; *P* <.001) and mother’s smoking during pregnancy (yes: 2.2% vs 6.0%; *P* = .01). In VDAART, significant differences were present for antibiotic prescriptions to the child at age 0-3 years (yes: 82% vs 55%; *P* <.001) and passive smoking exposure at age 0-1 year (yes: 7.7% vs 2.3%; *P* = .001) (Tables E2 and E3 in this article’s Online Repository at www.jacionline.org).

### Environmental exposures and urinary eicosanoids

A range of pre- and postnatal environmental exposures were investigated as determinants of the urinary eicosanoid profile in COPSAC_2010_ and VDAART (see Figs E1 and E2 in this article’s Online Repository at www.jacionline.org). Subsequently, we included all nominally significant determinants (*P* < .05) as covariates in the multivariable models in each cohort independent of their effect estimates.

### Eicosanoids at age 1 year and atopic disease in COPSAC_2010_

#### Wheeze/asthma.

Cross-sectional analyses showed that higher levels of the IsoP metabolite 2,3-dinor-8-iso-PGF_2α_ and TXA_2_ pathway metabolite 11-dehydro-2,3-dinor-TXB_2_ were associated with an increased risk of recurrent wheeze at 1 year (*P* < .05), but they did not pass FDR5%. Additionally, there were no significant associations for other individual or combined eicosanoid metabolites in the cross-sectional analyses.

**FIG 3.**
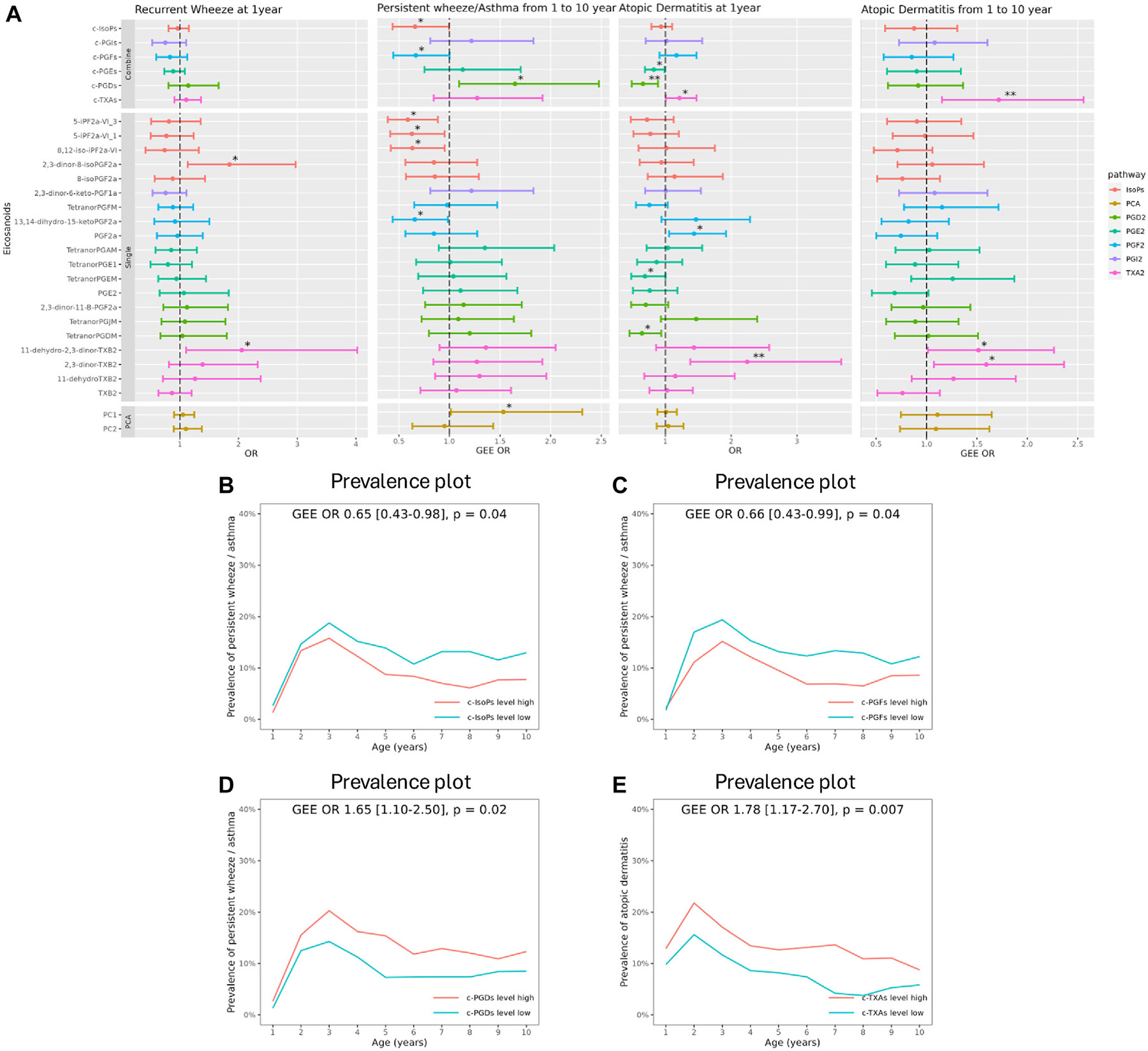
**A,** Associations between the urinary eicosanoids at age 1 year and risk of recurrent wheeze, persistent wheeze/asthma, and AD in COPSAC_2010_. For combined and single metabolites: *P < .05, **FDR < 0.05. For PC1 and PC2: *P < .05, **P < .01. **B-E,** Prevalence plot of persistent wheeze/asthma and AD in children divided into high/low metabolites level, that is, above versus below the median for c-IsoPs, c-PGFs, c-PGDs, c-TXAs in COPSAC_2010_. GEE, General estimation equation; *OR*, odds ratio.

Conversely, higher c-IsoPs and higher c-PGF_2_s were associated with a decreased risk of developing persistent wheeze/asthma from 1-10 years, whereas higher c-PGD_2_s were associated with an increased risk of persistent wheeze/asthma (*P* < .05). Further, higher levels of individual eicosanoid metabolites belonging to the IsoP and PGF_2_ pathways were also associated with a decreased risk of persistent wheeze/asthma, but neither of the individual or combined eicosanoid associations passed FDR5% (Fig 3, A, and Table E4 in this article’s Online Repository at www.jacionline.org). The yearly prevalence of persistent wheeze/asthma until age 10 years in children with levels of c-IsoPs, c-PGF2s, and c-PGD2s above versus below the median is shown in Fig 3, B-D.

#### Atopic dermatitis.

Cross-sectional analyses showed associations between higher levels of c-PGE_2_s and c-PGD_2_s and decreased risk of having AD (n = 51) at 1 year, whereas higher c-TXA_2_s were associated with an increased risk of AD, but only c-PGD_2_s passed FDR5%. The individual eicosanoid metabolites belonging to these subpathways showed similar associations, which passed FDR5% for the TXA_2_ pathway metabolite 2,3-dinor-TXB_2_ (Fig 3, A, Table E5 in this article’s Online Repository at www.jacionline.org).

Similarly, higher c-TXA_2_s (*P* < FDR5%) as well as the individual TXA_2_ pathway metabolites 11-dehydro-2,3-dinor-TXB_2_ and 2,3-dinor-TXB_2_ (*P* < .05) were associated with an increased risk of developing AD at 1-10 years. The yearly prevalence of AD until 10 years in children with levels of c-TXA_2_s above versus below the median is shown in Fig 3, E showing a persistent effect on AD.

#### Type 2 inflammation biomarkers.

Higher levels of c-TXA_2_s were positively associated with allergic sensitization and increasing Feno (ie, signs of type 2 inflammation). Further, higher individual eicosanoid metabolites from the TXA_2_ and IsoP pathways were associated with allergic sensitization, increasing blood eosinophil count, and Feno, but did not pass FDR5% (Fig E3 in this article’s Online Repository at www.jacionline.org).

#### PCA analysis.

A multivariate PCA model was performed in COPSAC_2010_, applied to investigate the association between the profile of eicosanoids and clinic end points, which showed that 20.4% of the variation was explained by PC1, reflecting increasing PGs and TXA_2_s and decreasing IsoPs, and 11.9% of the variation was explained by PC2, reflecting increasing TXA_2_s and decreasing PGE_2_s (Fig E4 in this article’s Online Repository at www.jacionline.org). Increasing PC1 was only significantly associated with an increased risk of developing persistent wheeze/asthma at 1-10 years (*P* < .05) (Fig 3, A).

### Eicosanoids at age 3 years and atopic disease in VDAART

#### Wheeze/asthma.

**FIG 4.**
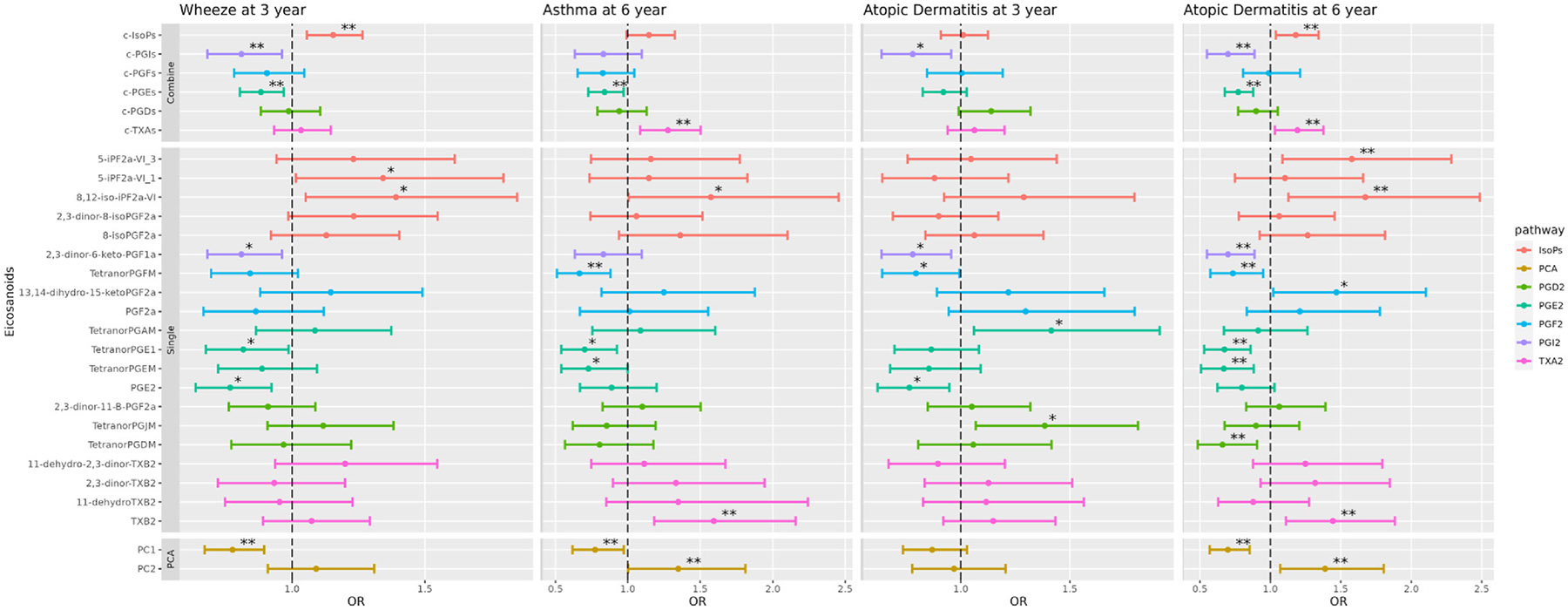
Associations between levels of urinary eicosanoids at age 3 years and risk of wheeze/asthma and AD in VDAART. For combined and single metabolites: *P < .05, **FDR < 0.05. For PC1 and PC2: *P < .05, **P < .01.

**FIG 5.**
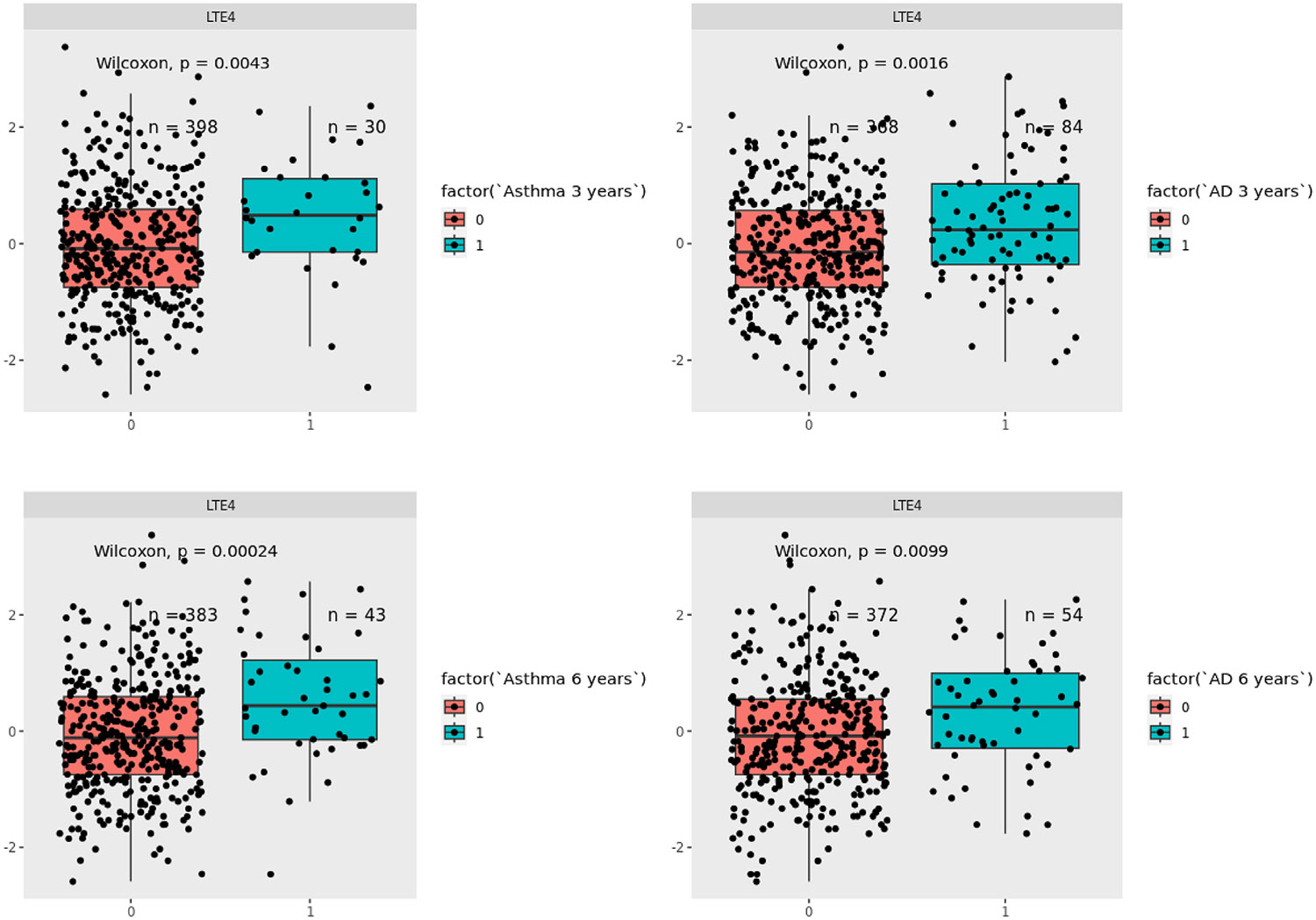
Associations between levels of urinary LTE_4_ at age 3 years and risk of asthma and AD in VDAART.

There was a cross-sectional association between higher levels of c-IsoPs and increased risk of wheeze at 3 years, whereas higher c-PGI_2_s and c-PGE_2_s were associated with a lower risk of wheeze, which all passed FDR5% significance. Individual metabolites in these subpathways showed similar associations with wheeze. For asthma at 6 years, higher c-PGE2s were associated with a decreased risk (*P* < FDR5%), whereas higher c-TXA_2_s were associated with a higher risk (*P* < FDR5%) with similar findings for several individual PGE_2_ and TXA_2_ pathway metabolites (Fig 4, Table E6 in this article’s Online Repository at www.jacionline.org). In addition, higher level of leukotriene E_4_ (LTE_4_) was associated with wheeze/asthma at 3 years (*P* < .01) and 6 years (*P* < .01) (Fig 5).

#### Atopic dermatitis.

The cross-sectional analyses of AD at 3 years only showed nominal association between higher c-PGI_2_s and lower risk, whereas there were many strong associations between urinary eicosanoid levels at 3 years and AD at 6 years. Like COPSAC_2010_, higher levels of c-TXA_2_s were associated with an increased risk of AD at 6 years, which passed FDR5%. In addition, higher c-IsoPs were also associated with an increased risk of AD, whereas higher c-PGI_2_s and c-PGE_2_s were associated with a lower risk (all *P* < FDR5%). Many individual metabolites in these subpathways showed similar associations passing FDR5% (Fig 4, Table E7 in this article’s Online Repository at www.jacionline.org). In addition, higher level of LTE_4_ was associated with increased risk of AD at 3 and 6 years (*P* < .01) (Fig 5).

#### Type-2 inflammation biomarkers.

Higher levels of 2,3-dinor-6-keto-PGF_1α_, tetranor PGFM, and PGE_2_ were all negatively associated with allergic sensitization but did not pass FDR5% (Fig E5 in this article’s Online Repository at www.jacionline.org). Further, higher levels of LTE_4_ were positively associated with allergic sensitization and increasing total-IgE (*P* < .05) (Fig E6 in this article’s Online Repository at www.jacionline.org).

#### PCA analysis.

The PC1 and PC2 in VDAART were calculated based on the PCA model of COPSAC_2010_. Decreasing PC1 was associated with an increased risk of wheeze at age 3 years (*P* < .001), asthma at 6 years (*P* < .001), AD at 6 years (*P* < .001), and also associated with an increased risk of allergic sensitization at age 6 years (*P* < .05). Increasing PC2 was associated with an increased risk of both asthma and AD at age 6 years (*P* <.001) (Fig 4).

## DISCUSSION

We investigated associations between urinary eicosanoid levels in early childhood and the development of atopic disease among 2 large mother–child cohorts with >1000 urine samples. In both cohorts, levels of urinary eicosanoids from various subpathways were associated with the development of wheeze, asthma, and AD. The most consistent finding was the FDR significant association between early life elevated levels of TXA_2_ subpathway metabolites and the risk of developing AD in both cohorts. Further, elevated urinary TXA_2_s in early life were also associated with the development of type-2 inflammation. These findings provide pathophysiological insight into the inception of atopic disease and type-2 inflammation.

In the cross-sectional analysis at 1 year in COPSAC_2010_, higher combined levels of urinary TXA_2_s were associated with an increased risk of AD. Furthermore, higher combined TXA_2_s at age 1 year and several metabolites in the TXA_2_ subpathway were associated with an increased risk of developing AD at 1-10 years. Similarly, higher TXA_2_s at 3 years in VDAART were also associated with an increased risk of AD at 6 years after multiple test corrections. The TXA_2_s are potent vasoconstrictors, reflecting greater platelet activation status.^27^ Platelets have long been recognized for their role in hemostasis and thrombosis,^28^ but studies suggested that platelets also have immune modulatory functions,^29^ and studies have further shown that platelets aggregation may contribute to the pathogenesis of AD.^30-32^ Previous cross-sectional studies have shown that both lesional and perilesional skin contain elevated levels of eicosanoids compared to uninvolved skin in individuals with AD,^33^ but our study is the first to report elevated urinary TXA_2_s in early life in relation to subsequent development of AD. In AD, TXA_2_ induced vasoconstriction and platelet aggregation, leading to decreased blood flow, may contribute to impaired skin perfusion and tissue damage.^34-36^

PGI_2_ inhibits platelet aggregation and acts as physiological antagonists of TXA_2_.^37^ This is in accordance with our results in VDAART where higher PGIs and particularly higher 2,3-dinor-6-keto-PGF_1_a at 3 years was associated with a decreased risk of AD at 6 years and further showed association in the cross-sectional analysis of AD at 3 years.

In the cross-sectional analysis in COPSAC_2010_, higher c-PGD_2_s associated with a decreased risk of AD at 1 year after multiple test correction with a similar nominal association for higher tetranor PGDM, which is the most abundant PGD_2_ pathway metabolite.^38^ In COPSAC_2010_, there were no associations between PGDs and subsequent risk of AD, whereas higher tetranor PGDM at 3 years in VDAART was associated with a decreased risk of AD at 6 years after multiple test corrections. Thus, the direction of association was consistent across cohorts and the differences observed in cross-sectional versus longitudinal analysis is probably due to the different ages of PGD_2_ measurements in the cohorts. PGD_2_ plays a multifaceted role in the pathophysiology of AD, contributing to inflammation, pruritus, immune dysregulation, and skin barrier dysfunction.^39^ PGD_2_ is the main PG produced by mast cells in AD and acts on 2 different receptors: D-prostanoid receptor 1 (DP1) and chemoattractant receptor-homologous molecule expressed on T_H_2 cells (CRTH2). It is thought that these receptors have contrasting effects in AD, with PGD_2_-DP1 signaling acting to reduce inflammation and preserve barrier function whereas PGD_2_-CRTH2 signaling acts to induce chemotaxis in leukocytes and promotes an inflammatory response.^40,41^ Although the important role of PGD_2_ in AD is evident, there is limited research in the role of early life PGD_2_ in the development of childhood AD.

There were 3 additional significant associations with AD after multiple test corrections in VDAART. First, higher c-PGE_2_s, tetranor PGE_1_, and tetranor PGEM associated with decreased risk of AD at 6 years. Similarly, higher c-PGE_2_s and tetranor PGEM at 1 year were nominally associated with decreased risk of AD in COPSAC_2010_ in the cross-sectional analyses. PGE_2_ is known for its dual effects as both a potent proinflammatory and vasodilatory,^42^ but also as stabilizer of mast cells. Previous studies showed increased PGE_2_ in the skin of adult patients with AD compared with of healthy patients.^33^ We note that the children in COPSAC_2010_ and VDAART developing AD had lower urinary PGE_2_s, suggesting a reduced immune protection. Second, higher IsoPs associated with increased risk of AD. IsoPs are considered key markers of systemic oxidative stress, and higher 8-IsoP has been shown in the skin of patients with AD,^14,43^ which aligns with our findings. Third, higher LTE_4_ associated with increased risk of AD, which is consistent with studies of LTE_4_ in adults with AD.

The metabolites belonging to the TXA_2_, PGI_2_, PGE_2_, and IsoP pathways, associated with AD and with wheeze/asthma. First, metabolites belonging to the TXA_2_ pathway associated with wheeze/asthma in both COPSAC_2010_ and VDAART. Higher levels of c-TXA_2_s and TXB_2_ associated with increased risk of asthma at 6 years in VDAART after multiple test corrections, while only a nominal association with recurrent wheeze at 1 year and 11-dehydro-2,3-dinor-TXB_2_ was observed in COPSAC_2010_. These findings are in line with studies suggesting a potential role of platelets in various lung diseases.^44,45^ Like AD, a similar pattern where higher 2,3-dinor-6-keto-PGF_1_a was associated with a decreased risk of wheeze at 3 years in VDAART.

Furthermore, in VDAART, lower levels of c-PGE_2_s were associated with wheeze at age 3 years and asthma at age 6 years after multiple test corrections, and the PGE_2_ and its downstream metabolites tetranor PGEM and tetranor PGE_1_ showed similar nominal associations. In line with this, a previous study showed lower urine levels of PGE_2_ and tetranor PGEM in adults with mild-to-moderate asthma.^3^ PGE_2_ is a dominant cyclooxygenase product of airway epithelium and smooth muscle and is considered to be immunomodulatory and predominantly bronchoprotective.^46^ The concentration of PGE_2_ influences platelet activation, with higher concentrations inhibiting activation and lower concentrations potentiating it.^47^ PGE_2_ inhibits both exercise-induced bronchoconstriction and allergen-induced early and late asthmatic responses^48,49^ and also prevents aspirin-induced bronchoconstriction in aspirin-sensitive asthma.^50^ Therefore, it is plausible that lower PGE_2_ could be associated with an increased risk of wheeze/asthma. Our study supports this and corroborates previous finding suggesting a protective role of PGE_2_s in young children for the development of wheeze and asthma in childhood. In addition, LTE_4_, a well-studied biomarker of asthma in adults and adolescents,^3^ was also increased in both 3- and 6-year-old children with asthma in VDAART.

In COPSAC_2010_ and VDAART, there were opposite associations with respect to IsoPs: IsoP associated (1) with an increased risk of wheeze at age 3 in the cross-sectional analysis in VDAART after multiple test correction and (2) with a similar nominal increased risk in COPSAC_2010_ in the 1 year cross-sectional analysis for the 2,3-dinor-8-*iso*-PGF_2ɑ_ IsoP metabolite. In contrast, increased c-IsoPs and several individual metabolites from that pathway were associated with a decreased risk of developing persistent wheeze/asthma at 1-10 years in COPSAC_2010_, but did not pass FDR5%. IsoPs are commonly referred to as markers of systemic oxidative stress, and higher levels of 8-IsoP in blood have been associated with asthma in a previous study of adults,^3,14^ and 8-IsoP concentration in exhaled breath condensate was significantly elevated in asthmatic children.^51,52^ Therefore, it is possible that the opposite findings for developing persistent wheeze/asthma in COPSAC_2010_ is either a low confidence finding or that IsoPs play a different role in early life prior to disease development.

The presence of shared associations between metabolites of the TXA_2_ and IsoP subpathways and AD and wheeze/asthma in both cohorts constitutes a molecular denominator of these conditions. Thus, these specific eicosanoids may be involved in common underlying mechanisms or pathways that contribute to the development of both AD and wheeze/asthma in early life and implies a potential interaction between skin and airway inflammation. Higher levels of TXA_2_s associated with allergic sensitization, blood eosinophils, and F_eno_ in COPSAC_2010_, which suggests a link to type-2 inflammation that is a common pathway in some subtypes of AD and asthma.^53^ In VDAART, we observed that increased LTE_4_ was associated with allergic sensitization and increasing total IgE, which validates the association between urinary LTE_4_ and type-2 inflammation biomarkers in a previous study of adults and adolescents^3^ and further proposes a role of LTE_4_ in the inception of type-2 inflammation in early childhood. However, the associations between TXA_2_ and LTE_4_ and type-2 biomarkers were not FDR significant, which may be due to the cohorts mainly consisting of healthy children.

This study has several strengths. First, a large number of urine samples was collected in early life prior to onset of most atopic disease, and samples were analyzed at the same laboratory using state-of-the-art methodology with a high reported detection rate of most eicosanoids. Second, we use longitudinal birth cohorts with prospective, comprehensive, and carefully collected clinical data obtained from population-based cohorts. Third, we benefit from evidence derived from 2 independent cohorts allowing for stronger interpretations of findings that were present in both.

This study also has 7 limitations. (1) One limitation is that the racial composition of the 2 cohorts differs with COPSAC_2010_ predominantly being White and VDAART being multiracial. (2) COPSAC_2010_ is population-based and VDAART a high-risk cohort. (3) There are differences in baseline characteristics among children with and without eicosanoid data in the 2 cohorts: COPSAC (sex, mother’s smoking during pregnancy) and VDAART (antibiotic prescriptions, passive smoking exposure). (4) The different measuring time points in the cohorts—age 1 year versus age 3 years—may partly explain the discrepancies in findings across cohorts as atopic diseases may both develop and vanish in this time frame. (5) It is also a limitation that we do not have serial measurements of eicosanoids in the cohorts. (6) The concentrations of LTE_4_ had >30% missing values in COPSAC_2010_ and were therefore not included in the analysis. (7) Parental reporting is a nonreliable diagnosis and has the potential of error due to recall bias in VDAART.

This exploratory study in 2 independent cohorts demonstrates that early life perturbations in the eicosanoid pathways may herald the onset of atopic disease and type-2 inflammation in childhood, which provides novel pathophysiological insight in disease inception.

## Supplementary Material

Supplementary Information

Supplementary Figure 2

Supplementary Figure 1

Supplementary Figure 3

Supplementary Figure 5

Supplementary Figure 4

Supplementary Figure 6

## Clinical implications:

Early life perturbations in the eicosanoid pathways measured in urine samples may predict later atopic disease risk and provide pathophysiological insight in the inception of atopic diseases.

## Abbreviations used

AD: Atopic dermatitis
c-TXA_2_s: Combined TXA_2_s
FDR5%: False discovery rate of 5%
Feno: Fractional exhaled nitric oxide
IsoPs: Isoprostanes
LTE_4_: Leukotriene E_4_
LTs: Leukotrienes
PCA: Principal component analysis
PGs: Prostaglandins
PGD_2_: Prostaglandin D_2_
TXs: Thromboxanes
TXA_2_: Thromboxane A_2_
