# Supplementary material for: Urinary eicosanoid levels in early life and risk of atopic disease in childhood
Source: J Allergy Clin Immunol. Author manuscript; Available in PMC 2025 Apr 30. (PMC12042789; doi:10.1016/j.jaci.2024.05.022)
Supplement: Supplementary Information [file NIHMS2072188-supplement-Supplementary_Information.docx]

**Supplementary Information**

**Title**:

Urinary eicosanoid levels in early life and risk of atopic disease in childhood

**Authors**:

Liang Chen, MSc^1+3^; Nicklas Brustad, MD, PhD^1^; Min Kim, MSc, PhD^1+2^; Yang Luo, MSc^1^; Tingting Wang, MSc^1^, PhD; Mina Ali, MSc, PhD^1^; Nicole Prince, BSc, PhD^3^; Yulu Chen, BSc, PhD^3^; Su Chu, MD, PhD^3^; Sofina Begum, BSc, PhD^3^; Kevin Mendez, BSc, PhD^3^; Rachel S. Kelly, BSc, PhD^3^; Ann-Marie Schoos, MD, PhD^1+4^; Morten A. Rasmussen, MSc, PhD^1+5^; Javier Zurita MSc, PhD^6+7^; Johan Kolmert, MSc, PhD^6+7^; Jakob Stokholm, MD, PhD^1+4+5^; Augusto Litonjua, MD, MPH^8^; Scott T. Weiss, MD, MS^3^; Klaus Bønnelykke, MD, PhD^1^; Craig E. Wheelock, MSc, PhD^6+7*^; Jessica Lasky-Su, ScD^3*^; Bo Chawes, MD, PhD, DMSc^1*^.

Data Collection

**Secondary Clinical Endpoints: COPSAC_2010_**

Type-2 inflammation biomarkers at age 6 years:

*Allergic sensitization*: Serum specific-IgE levels were measured against a panel of common inhaled and food allergens (ImmunoCAP Phadiatop Infant™ and ImmunoCAP Phadiatop™, Thermo Fisher Scientific, Uppsala, Sweden). Sensitization was defined as any specific-IgE ≥ 0.35 kUA/L.

*Fraction of exhaled nitric oxide (FeNO)*: FeNO was measured in duplicates by an online technique using NIOX Vero (Aerocrine AB, Solna, Sweden) in accordance with international guidelines.

*Blood eosinophil count*: Peripheral blood eosinophil count was determined as an absolute count (10^9 cells/L) using ADVIA® 2120i Hematology System, Siemens Healthcare Diagnostics Inc., Munich, Germany, and Sysmex XE, Sysmex Nordic Aps, Denmark.

**Secondary Clinical Endpoints: VDAART**

Type-2 inflammation biomarkers: Allergic sensitization status and total-IgE measurements were available at age 6 years.

**Quantification of Urinary Eicosanoids**

Urine samples were randomly divided into batches of 82 samples of each, comprising a total 13 batches. Eicosanoids were quantified in extracted urine by liquid chromatography coupled to tandem mass spectrometry (LC-MS/MS) method using an external calibration curve, quality control samples and appropriate deuterated internal standards. Concentrations were normalized by specific gravity.

Data Analysis

Among the 21 metabolites representing six key pathways, leukotriene E_4_ (LTE_4_) had more than 30% missing values in the COPSAC_2010_ study and was excluded for further analysis there and only analyzed in VDAART (21% missing values). Values below detection level were imputed with half of the minimum value for all eicosanoids. Prior to statistical analyses, all metabolite levels were log-transformed for normality and z-scored for comparability of estimates across eicosanoids.

**Equation of association tests**

Linner regression: lm(metabolite ~ environmental exposure, data)

Logistic regression: glm(disease ~ metabolite + covariates, family=binomial, data)

Cox regression: coxph(Surv (eventage, event) ~ metabolite + covariates, data)

GEE OR model: geeglm(curr_disease ~ factor(period) + factor(predictor), id=abcno, data=modeldate, family = binomial(link="logit"), corstr="independence")

**eTable 1**: The sub-pathway classification and abbreviation of eicosanoid metabolites.

| **Category** | **Sub Pathway** | **Combine Metabolites** | **Individual Metabolites** |
| --- | --- | --- | --- |
| TXA2s | TXA2 | c-TXA2s | TXB_2_ |
|  |  |  | 11-dehydro-TXB_2_ |
|  |  |  | 2,3-dinor-TXB_2_ |
|  |  |  | 11-dehydro-2,3-dinor-TXB_2_ |
| PGD2s | PGD2 | c-PGD2s | TetranorPGDM |
|  |  |  | TetranorPGJM |
|  |  |  | 2,3-dinor-11-β-PGF_2a_ |
| PGE2s | PGE2 | c-PGE2s | PGE_2_ |
|  |  |  | TetranorPGEM |
|  |  |  | TetranorPGE_1_ |
|  |  |  | TetranorPGAM |
| PGF2s | PGF2 | c-PGF2s | TetranorPGFM |
|  |  |  | PGF_2α_ |
|  |  |  | 13,14-dihydro-15-ketoPGF_2α_ |
| PGI2s | PGI2 | c-PGI2s | 2,3-dinor-6-keto-PGF_1a_ |
| Isoprostanes | IsoPs | c-IsoPs | 8-iso-PGF_2a_ |
|  |  |  | 2,3-dinor-8-iso-PGF_2a_ |
|  |  |  | 5-iPF_2α_-VI_1_ |
|  |  |  | 5-iPF_2α_-VI_3_ |
|  |  |  | 8,12-iso-iPF_2a_-VI |

**eTable 2**: Comparison of baseline characteristics among children with vs. without eicosanoids data in COPSAC_2010_.

| **Characteristic** | **N** | **Overall**, N = 698*^1^* | **Eicosanoids test: No**, N = 249*^1^* | **Eicosanoids test: Yes**, N = 449*^1^* | **p-value***^2^* |
| --- | --- | --- | --- | --- | --- |
| n-3 LCPUFA RCT | 698 |  |  |  | 0.8 |
| n-3 LCPUFA |  | 347 (50%) | 125 (50%) | 222 (49%) |  |
| Placebo |  | 351 (50%) | 124 (50%) | 227 (51%) |  |
| Sex | 698 |  |  |  | <0.001 |
| Female |  | 340 (49%) | 157 (63%) | 183 (41%) |  |
| Male |  | 358 (51%) | 92 (37%) | 266 (59%) |  |
| Race | 698 |  |  |  | 0.094 |
| Caucasian |  | 668 (96%) | 234 (94%) | 434 (97%) |  |
| Non-caucasian |  | 30 (4.3%) | 15 (6.0%) | 15 (3.3%) |  |
| Vitamin D RCT | 587 |  |  |  | 0.6 |
| Placebo |  | 289 (49%) | 94 (48%) | 195 (50%) |  |
| Vitamin D3 |  | 298 (51%) | 103 (52%) | 195 (50%) |  |
| Mother´s smoking pregnancy | 698 |  |  |  | 0.010 |
| Non-smoking |  | 673 (96%) | 234 (94%) | 439 (98%) |  |
| Smoking |  | 25 (3.6%) | 15 (6.0%) | 10 (2.2%) |  |
| Mother´s passive smoking pregnancy | 698 |  |  |  | 0.11 |
| Non-passive smoking |  | 595 (85%) | 205 (82%) | 390 (87%) |  |
| Passive smoking |  | 103 (15%) | 44 (18%) | 59 (13%) |  |
| Antibiotic usage during pregnancy | 697 | 254 (36%) | 92 (37%) | 162 (36%) | 0.8 |
| Delivery | 698 |  |  |  | 0.8 |
| Caesarean section |  | 150 (21%) | 55 (22%) | 95 (21%) |  |
| Normal |  | 548 (79%) | 194 (78%) | 354 (79%) |  |
| Season | 698 |  |  |  | 0.8 |
| Autumn |  | 149 (21%) | 50 (20%) | 99 (22%) |  |
| Spring |  | 186 (27%) | 65 (26%) | 121 (27%) |  |
| Summer |  | 149 (21%) | 58 (23%) | 91 (20%) |  |
| Winter |  | 214 (31%) | 76 (31%) | 138 (31%) |  |
| Serum EPA+DHA level at 6 Month | 563 | 2.60 (1.74, 3.98) | 2.46 (1.62, 3.74) | 2.72 (1.82, 4.14) | 0.071 |
| Serum Arachidic acid level at 6 Month | 563 | 1.25 (0.92, 1.79) | 1.21 (0.89, 1.73) | 1.26 (0.96, 1.83) | 0.2 |
| Serum Vitamin D level at 6 Month | 578 | 34 (28, 41) | 34 (28, 39) | 34 (28, 41) | 0.3 |
| Antibiotic to child | 695 | 10 (1.4%) | 3 (1.2%) | 7 (1.6%) | >0.9 |
| Exclusive breastfeeding, Days | 692 | 122 (46, 150) | 122 (43, 147) | 122 (54, 151) | 0.5 |
| Breastfeeding, Days | 691 | 235 (151, 322) | 222 (152, 318) | 242 (151, 327) | 0.4 |
| Children`s passive smoking 0-1year Days | 687 | 2 (0, 27) | 2 (0, 50) | 2 (0, 25) | 0.4 |
| Neonate hospitalized after birth | 698 |  |  |  | 0.3 |
| 0 |  | 618 (89%) | 216 (87%) | 402 (90%) |  |
| 1 |  | 80 (11%) | 33 (13%) | 47 (10%) |  |
| Income | 698 |  |  |  | 0.067 |
| High |  | 128 (18%) | 57 (23%) | 71 (16%) |  |
| Low |  | 60 (8.6%) | 21 (8.4%) | 39 (8.7%) |  |
| Medium |  | 510 (73%) | 171 (69%) | 339 (76%) |  |
| Maternal educational level | 698 |  |  |  | 0.5 |
| High |  | 205 (29%) | 79 (32%) | 126 (28%) |  |
| Low |  | 51 (7.3%) | 20 (8.0%) | 31 (6.9%) |  |
| Medium |  | 442 (63%) | 150 (60%) | 292 (65%) |  |

*^1^* n (%); Median (IQR)

*^2^* Pearson's Chi-squared test; Wilcoxon rank sum test; Fisher's exact test

**eTable 3**: Comparison of baseline characteristics among children with vs. without eicosanoids data in VDAART.

| **Characteristic** | **N** | **Overall**,  N = 880*^1^* | **Eicosanoids test: No**, N = 305*^1^* | **Eicosanoids test: Yes**, N = 575*^1^* | **p-value***^2^* |
| --- | --- | --- | --- | --- | --- |
| Vitamin D RCT | 806 |  |  |  | 0.3 |
| Placebo |  | 401 (50%) | 121 (52%) | 280 (49%) |  |
| Vitamin D3 |  | 405 (50%) | 110 (48%) | 295 (51%) |  |
| Sex | 816 |  |  |  | 0.3 |
| Female |  | 388 (48%) | 122 (51%) | 266 (46%) |  |
| Male |  | 428 (52%) | 119 (49%) | 309 (54%) |  |
| Race | 806 |  |  |  | 0.071 |
| Amer. Indian |  | 6 (0.7%) | 2 (0.9%) | 4 (0.7%) |  |
| Asian |  | 48 (6.0%) | 8 (3.5%) | 40 (7.0%) |  |
| Black, African American |  | 390 (48%) | 102 (44%) | 288 (50%) |  |
| Native Hawaiian |  | 12 (1.5%) | 6 (2.6%) | 6 (1.0%) |  |
| Other |  | 85 (11%) | 28 (12%) | 57 (9.9%) |  |
| White |  | 265 (33%) | 85 (37%) | 180 (31%) |  |
| Any smoking pregnancy | 841 | 18 (2.1%) | 2 (0.7%) | 16 (2.8%) | 0.056 |
| Delivery | 817 |  |  |  | 0.5 |
| Caesarean section |  | 241 (29%) | 67 (28%) | 174 (30%) |  |
| Normal |  | 576 (71%) | 175 (72%) | 401 (70%) |  |
| Birthweight | 812 | 3,300 (2,960, 3,640) | 3,325 (3,030, 3,665) | 3,285 (2,920, 3,624) | 0.074 |
| Serum Vitamin D level at 1 Year | 631 | 29 (24, 34) | 29 (25, 35) | 29 (24, 34) | 0.4 |
| Antibiotic to child 0-3 Years | 876 | 639 (73%) | 167 (55%) | 472 (82%) | <0.001 |
| Exclusive breastfeeding for 4 months | 738 | 247 (33%) | 63 (32%) | 184 (34%) | 0.6 |
| Children`s passive smoking 0-1 Year | 876 | 51 (5.8%) | 7 (2.3%) | 44 (7.7%) | 0.001 |
| Hospitalizations 0-3 year | 876 | 184 (21%) | 55 (18%) | 129 (22%) | 0.2 |
| Income | 806 |  |  |  | 0.8 |
| High |  | 93 (12%) | 24 (10%) | 69 (12%) |  |
| Low |  | 341 (42%) | 95 (41%) | 246 (43%) |  |
| Medium |  | 176 (22%) | 55 (24%) | 121 (21%) |  |
| NA |  | 196 (24%) | 57 (25%) | 139 (24%) |  |
| Maternal education level | 806 |  |  |  | 0.5 |
| College graduate or Graduate school |  | 273 (34%) | 83 (36%) | 190 (33%) |  |
| High school, Technical school |  | 241 (30%) | 70 (30%) | 171 (30%) |  |
| Less than high school |  | 100 (12%) | 22 (9.5%) | 78 (14%) |  |
| Some college |  | 192 (24%) | 56 (24%) | 136 (24%) |  |

*^1^* n (%); Median (IQR)

*^2^* Pearson's Chi-squared test; Fisher's exact test; Wilcoxon rank sum test

**eTable 4**: Associations between urinary eicosanoids at age 1 year and wheeze/asthma in COPSAC2010. *P<0.05, **FDR<0.05.

| **COPSAC 2010** | | | | | | | |
| --- | --- | --- | --- | --- | --- | --- | --- |
| **Pathway** | **Eicosanoids** | **Recurrent wheeze**  **at 1 year** | | | **Asthma**  **from 1 to 10 years** | | |
|  |  | **aOR** | **P.value** | **FDR** | **GEE OR** | **P.value** | **FDR** |
| TXA2 | 11-dehydro-2,3-dinor-TXB_2_ | 2.05[1.11-4.02] | **0.031*** | 0.301 | 1.36[0.90-2.05] | 0.142 | 0.511 |
| PGD2 | c-PGD2s | 1.14[0.81-1.66] | 0.470 | 0.627 | 1.64[1.09-2.48] | **0.0163*** | 0.130 |
| PGF2 | c-PGF2s | 0.83[0.59-1.13] | 0.257 | 0.511 | 0.67[0.44-1.00] | **0.050*** | 0.135 |
| IsoPs | 2,3-dinor-8-iso-PGF_2a_ | 1.85 [1.13-2.97] | **0.0121*** | 0.242 | 0.85 [0.56-1.27] | 0.426 | 0.706 |
|  | 8,12-iso-iPF_2a_-VI | 0.74[0.41-1.32] | 0.307 | 0.768 | 0.63[0.41-0.95] | **0.029*** | 0.192 |
|  | 5-iPF_2α_-VI_1_ | 0.77[0.50-1.24] | 0.246 | 0.768 | 0.63[0.41-0.95] | **0.029*** | 0.192 |
|  | 5-iPF_2α_-VI_3_ | 0.81[0.51-1.35] | 0.405 | 0.768 | 0.58[0.39-0.86] | **0.011*** | 0.192 |
|  | c-IsoPs | 0.96[0.80-1.15] | 0.688 | 0.688 | 0.66[0.43-0.99] | **0.047*** | 0.135 |
| PCA model | PC1 | 1.05 [0.90-1.24] | 0.544 |  | 1.53 [1.02-2.31] | **0.042*** |  |
|  | PC2 | 1.10 [0.89-1.37] | 0.375 |  | 0.95 [0.63-1.43] | 0.811 |  |

**eTable 5**: Associations between urinary eicosanoids at age 1 year and atopic dermatitis in COPSAC2010. *P<0.05, **FDR<0.05.

| **COPSAC 2010** | | | | | | | |
| --- | --- | --- | --- | --- | --- | --- | --- |
| **Pathway** | **Eicosanoids** | **Atopic dermatitis**  **at 1 year** | | | **Atopic dermatitis**  **from 1 to 10 years** | | |
|  |  | **aOR** | **P.value** | **FDR** | **GEE OR** | **P.value** | **FDR** |
| TXA2 | TXB_2_ | 1.03[0.76-1.42] | 0.870 | 0.966 | 0.76[0.51-1.13] | 0.177 | 0.506 |
|  | 11-dehydro-TXB_2_ | 1.15[0.68-2.05] | 0.620 | 0.827 | 1.26[0.85-1.88] | 0.242 | 0.565 |
|  | 2,3-dinor-TXB_2_ | 2.24[1.38-3.67] | **0.0011*** | **0.0227**** | 1.59[1.07-2.36] | **0.021*** | 0.408 |
|  | 11-dehydro-2,3-dinor-TXB_2_ | 1.44[0.86-2.58] | 0.203 | 0.372 | 1.51[1.01-2.26] | **0.043*** | 0.409 |
|  | c-TXA2s | 1.21[1.00-1.47] | **0.046*** | 0.123 | 1.71[1.15-2.56] | **0.007*** | 0.063 |
| PGD2 | TetranorPGDM | 0.64[0.45-0.94] | **0.015*** | 0.108 | 1.02[0.68-1.51] | 0.937 | 0.942 |
|  | c-PGD2s | 0.66[0.49-0.89] | **0.0058*** | **0.0467**** | 0.92[0.62-1.36] | 0.666 | 0.727 |
| PGE2 | TetranorPGEM | 0.69[0.48-1.00] | **0.046*** | 0.228 | 1.26[0.85-1.87] | 0.254 | 0.565 |
|  | c-PGE2s | 0.82[0.69-0.99] | **0.032*** | 0.123 | 0.90[0.61-1.34] | 0.609 | 0.727 |
| PGF2 | PGF_2a_ | 1.44 [1.06-1.92] | **0.016*** | 0.108 | 0.74 [0.50-1.11] | 0.143 | 0.506 |
| PCA model | PC1 | 1.01 [0.87-1.17] | 0.902 |  | 1.11 [0.75-1.64] | 0.613 |  |
|  | PC2 | 1.04 [0.87-1.28] | 0.661 |  | 1.09 [0.74-1.62] | 0.661 |  |

**eTable 6**: Associations between urinary eicosanoids at age 3 years and wheeze/asthma in VDAART. *P<0.05, **FDR<0.05.

| **VDAART** | | | | | | | | |
| --- | --- | --- | --- | --- | --- | --- | --- | --- |
| **Pathway** | **Eicosanoids** | **Wheeze**  **at 3 years** | | | **Asthma**  **at 6 years** | | | |
|  |  | **aOR** | **P.value** | **FDR** | **aOR** | **P.value** | **FDR** |  |
| TXA2 | TXB_2_ | 1.07[0.89-1.29] | 0.455 | 0.568 | 1.59[1.18-2.16] | **0.0023*** | **0.033**** |  |
|  | c-TXA2s | 1.03[0.93-1.15] | 0.528 | 0.604 | 1.28[1.09-1.50] | **0.0031*** | **0.024**** |  |
| PGE2 | PGE_2_ | 0.77[0.64-0.92] | **0.0049*** | 0.098 | 0.89[0.67-1.20] | 0.423 | 0.651 |  |
|  | TetranorPGEM | 0.89[0.72-1.09] | 0.225 | 0.413 | 0.73[0.54-0.99] | **0.039*** | 0.184 |  |
|  | TetranorPGE_1_ | 0.82[0.68-0.98] | **0.0353*** | 0.171 | 0.70[0.54-0.92] | **0.0093*** | 0.062 |  |
|  | c-PGE2s | 0.88[0.80-0.97] | **0.0088*** | **0.0234**** | 0.84[0.73-0.97] | **0.017*** | 0.069 |  |
| PGF2 | TetranorPGFM | 0.84[0.69-1.02] | 0.079 | 0.227 | 0.67[0.51-0.88] | **0.0033*** | **0.033**** |  |
| PGI2 | 2,3-dinor-6-keto-PGF_1a_ | 0.81 [0.68-0.96] | **0.016*** | 0.143 | 0.83[0.63-1.10] | 0.186 | 0.464 |  |
| IsoPs | 8,12-iso-iPF_2a_-VI | 1.39 [1.05-1.85] | **0.0215*** | 0.143 | 1.57[1.01-2.45] | **0.046*** | 0.184 |  |
|  | 5-iPF2a-VI_1_ | 1.34 [1.01-1.80] | **0.0427*** | 0.171 | 1.15[0.74-1.83] | 0.562 | 0.702 |  |
|  | c-IsoPs | 1.15 [1.06-1.27] | **0.0018*** | **0.0146**** | 1.15[0.99-1.32] | 0.065 | 0.172 |  |
| PCA model | PC1 | 0.78 [0.67-0.89] | **0.0005**** |  | 0.77 [0.62-0.97] | **0.026*** |  |  |
|  | PC2 | 1.09 [0.91-1.31] | 0.35 |  | 1.35 [1.00-1.81] | **0.047*** |  |  |

**eTable 7**: Associations between urinary eicosanoids at age 3 years and atopic dermatitis in VDAART. *P<0.05, **FDR<0.05.

| **VDAART** | | | | | | | |
| --- | --- | --- | --- | --- | --- | --- | --- |
| **Pathway** | **Eicosanoids** | **Atopic dermatitis**  **at 3 years** | | | **Atopic dermatitis**  **at 6 years** | | |
|  |  | **aOR** | **P.value** | **FDR** | **aOR** | **P.value** | **FDR** |
| TXA2 | TXB_2_ | 1.15[0.92-1.43] | 0.217 | 0.395 | 1.44[1.11-1.88] | **0.0062*** | **0.031**** |
|  | c-TXA2s | 1.06[0.94-1.20] | 0.327 | 0.654 | 1.19[1.03-1.38] | **0.017*** | **0.022**** |
| PGD2 | TetranorPGDM | 1.06[0.81-1.42] | 0.695 | 0.732 | 0.66[0.48-0.91] | **0.0088*** | **0.035**** |
|  | TetranorPGJM | 1.38[1.07-1-81] | **0.016*** | 0.732 | 0.90[0.68-1.21] | 0.467 | 0.584 |
| PGE2 | PGE_2_ | 0.77[0.62-0.95] | **0.013*** | 0.105 | 0.80[0.62-1.03] | 0.076 | 0.152 |
|  | TetranorPGEM | 0.85[0.68-1.09] | 0.193 | 0.395 | 0.67[0.51-0.88] | **0.0039*** | **0.026**** |
|  | TetranorPGE1 | 0.87[0.69-1.08] | 0.196 | 0.395 | 0.67[0.53-0.86] | **0.0013*** | **0.025**** |
|  | TetranorPGAM | 1.41[1.06-1.91] | **0.021*** | 0.105 | 0.91[0.67-1.26] | 0.575 | 0.676 |
|  | c-PGE2s | 0.92[0.83-1.03] | 0.138 | 0.367 | 0.77[0.68-0.88] | **0.00009*** | **0.0007**** |
| PGF2 | 13,14-dihydro-15-ketoPGF_2a_ | 1.22[0.89-1.66] | 0.212 | 0.395 | 1.47[1.02-2.10] | **0.037*** | 0.081 |
|  | TetranorPGFM | 0.79[0.64-0.99] | **0.040*** | 0.162 | 0.73[0.57-0.95] | **0.015*** | **0.039**** |
| PGI2 | 2,3-dinor-6-keto-PGF_1a_ | 0.78[0.64-0.96] | **0.017*** | 0.105 | 0.70[0.55-0.89] | **0.0033*** | **0.026**** |
| IsoPs | 8,12-iso-iPF_2a_-VI | 1.29[0.92-1.80] | 0.134 | 0.383 | 1.67[1.13-2.49] | **0.010*** | **0.035**** |
|  | 5-iPF2a-VI_3_ | 1.05[0.76-1.44] | 0.779 | 0.779 | 1.58[1.09-2.28] | **0.016*** | **0.039**** |
|  | c-IsoPs | 1.01[0.91-1.12] | 0.831 | 0.956 | 1.18[1.04-1.34] | **0.011*** | **0.022**** |
| PCA model | PC1 | 0.97[0.78-1.21] | 0.782 |  | 0.69[0.57-0.85] | **0.0004**** |  |
|  | PC2 | 0.87 [0.74-1.03] | 0.102 |  | 1.39 [1.07-1.80] | **0.0134**** |  |

**eFigure 1**: Overview of associations between environmental exposures and urinary eicosanoids at age 1 year in COPSAC_2010_. *P<0.05, **FDR<0.05.

**eFigure 2**: Overview of associations between environmental exposures and urinary eicosanoids at 3 years in VDAART. *P<0.05, **FDR<0.05.

**eFigure 3**: Associations between the urinary eicosanoids at age 1 year and Type 2 inflammation biomarkers at age 6 years in COPSAC_2010_. *P<0.05, **FDR<0.05.

**eFigure 4**: PCA loading plot of urinary eicosanoids in COPSAC_2010_.

**eFigure 5**: Associations between the urinary eicosanoids at age 3 years and Type 2 inflammation biomarkers at age 6 years in VDAART. *P<0.05, **FDR<0.05.

**eFigure 6**: Associations between levels of urinary LTE_4_ at age 3 years and Type-2 inflammation biomarkers at age 6 years in VDAART. *P<0.05, **FDR<0.05.
