# Supplementary figures and images for: Urinary eicosanoid levels in early life and risk of atopic disease in childhood
Source: J Allergy Clin Immunol. Author manuscript; Available in PMC 2025 Apr 30. (PMC12042789; doi:10.1016/j.jaci.2024.05.022)

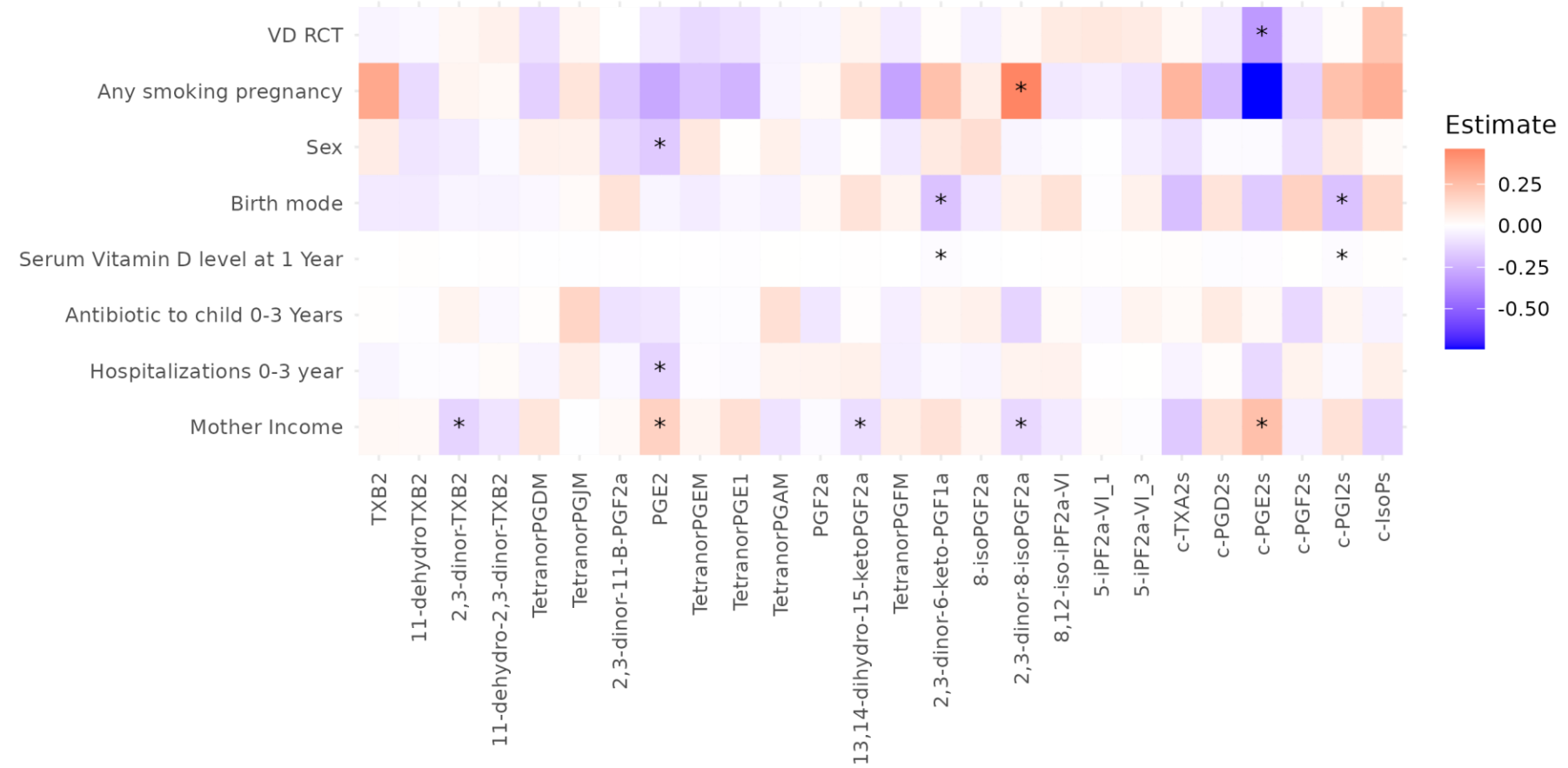

Supplement: Supplementary Figure 2 [file NIHMS2072188-supplement-Supplementary_Figure_2.pdf]

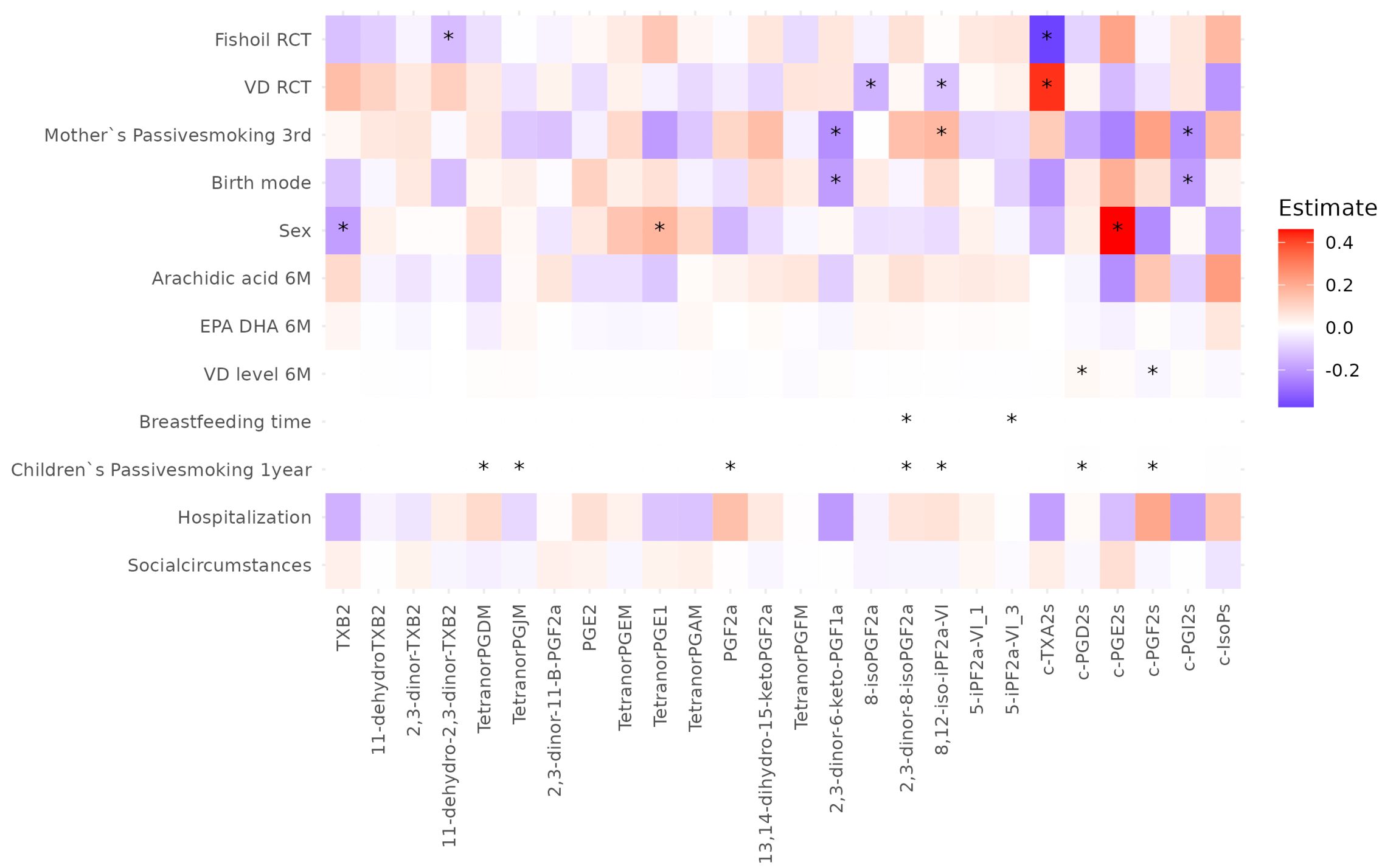

Supplement: Supplementary Figure 1 [file NIHMS2072188-supplement-Supplementary_Figure_1.pdf]

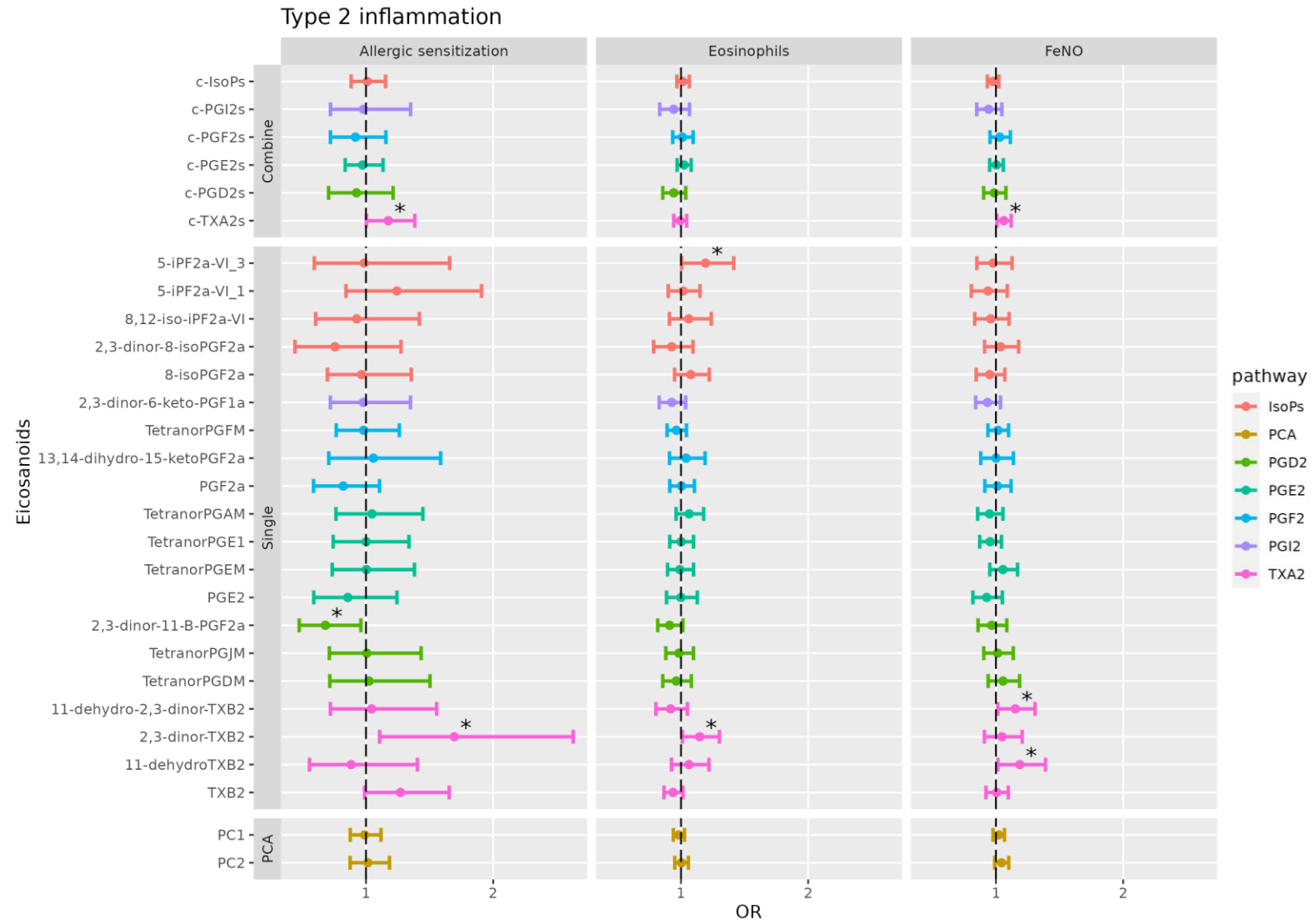

Supplement: Supplementary Figure 3 [file NIHMS2072188-supplement-Supplementary_Figure_3.pdf]

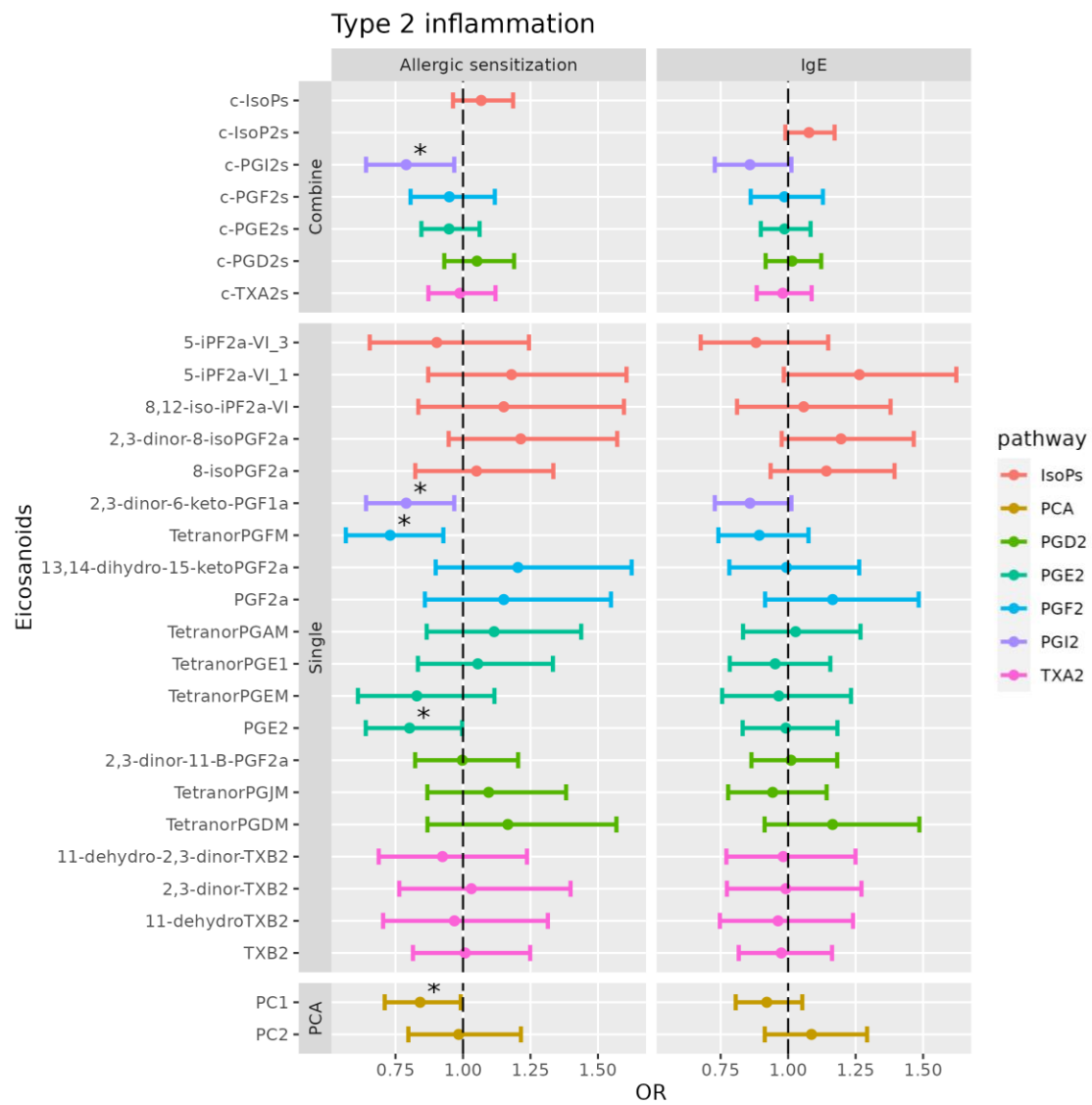

Supplement: Supplementary Figure 5 [file NIHMS2072188-supplement-Supplementary_Figure_5.pdf]

PCA - Biplot

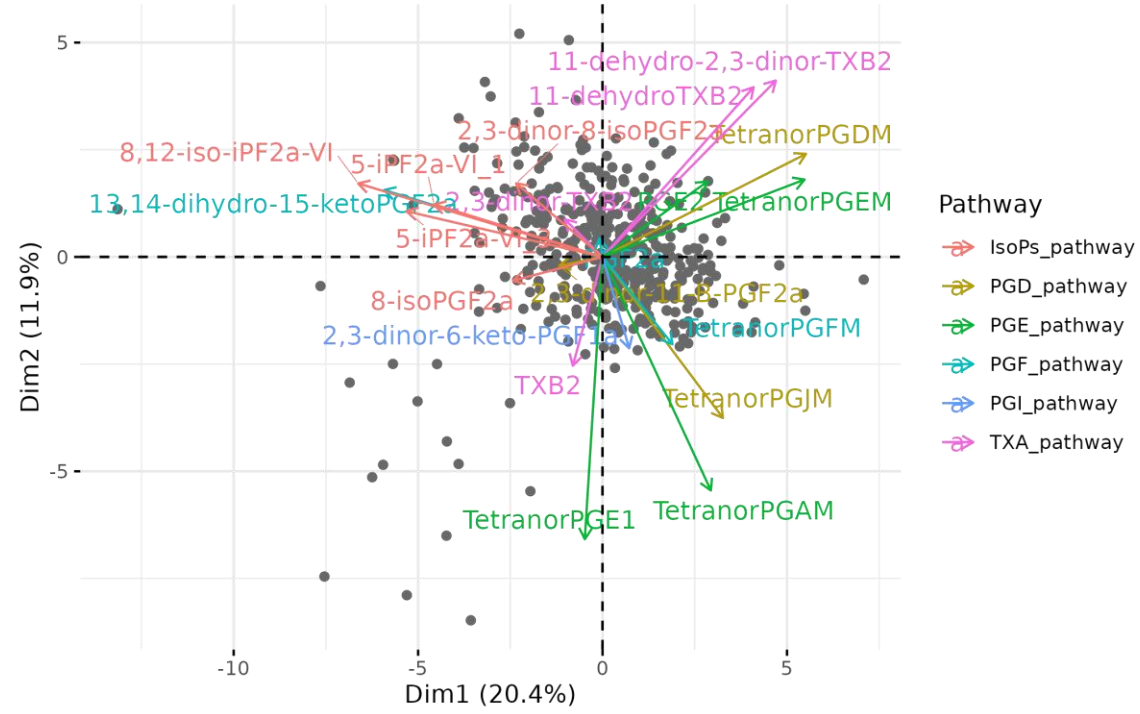

Supplement: Supplementary Figure 4 [file NIHMS2072188-supplement-Supplementary_Figure_4.pdf]

### LTE4 related Type 2 inflammation

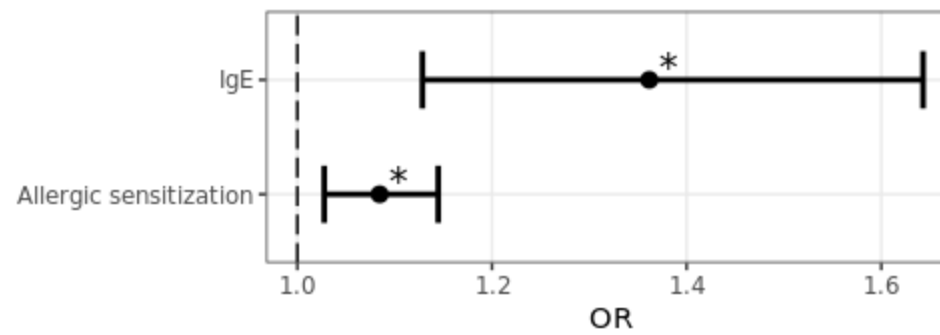

Supplement: Supplementary Figure 6 [file NIHMS2072188-supplement-Supplementary_Figure_6.pdf]
